# Expression of Growth Hormone-Releasing Hormone and Its Receptor Splice Variants in Primary Human Endometrial Carcinomas: Novel Therapeutic Approaches

**DOI:** 10.3390/molecules27092671

**Published:** 2022-04-21

**Authors:** Zsuzsanna Szabo, Eva Juhasz, Andrew V. Schally, Balazs Dezso, Sandor Huga, Zoltan Hernadi, Gabor Halmos, Csongor Kiss

**Affiliations:** 1 Department of Biopharmacy, Faculty of Pharmacy, University of Debrecen, 4032 Debrecen, Hungary; szabo.zsuzsanna@pharm.unideb.hu (Z.S.); halmos.gabor@pharm.unideb.hu (G.H.); 2 Department of Pediatrics, Faculty of Medicine, University of Debrecen, 4032 Debrecen, Hungary; juhasze@med.unideb.hu; 3 Veterans Affairs Medical Center, Endocrine, Polypeptide and Cancer Institute, Miami, FL 33125, USA; andrew.schally@va.gov; 4 Department of Pathology, Department of Medicine, Divisions of Hematology-Oncology and Endocrinology, Miller School of Medicine, University of Miami, Miami, FL 33101, USA; 5 Sylvester Comprehensive Cancer Center, University of Miami, Miami, FL 33136, USA; 6 Department of Pathology, Faculty of Medicine, University of Debrecen, 4032 Debrecen, Hungary; bdezso@med.unideb.hu; 7 Department of Obstetrics and Gynecology, Faculty of Medicine, University of Debrecen, 4032 Debrecen, Hungary; sandor.huga@ppd.com (S.H.); hz@med.unideb.hu (Z.H.)

**Keywords:** GHRH, receptors for GHRH, splice variants, human endometrial carcinoma

## Abstract

Antagonists of growth hormone-releasing hormone (GHRH) inhibit the growth of various tumors, including endometrial carcinomas (EC). However, tumoral receptors that mediate the antiproliferative effects of GHRH antagonists in human ECs have not been fully characterized. In this study, we investigated the expression of mRNA for GHRH and splice variants (SVs) of GHRH receptors (GHRH-R) in 39 human ECs and in 7 normal endometrial tissue samples using RT-PCR. Primers designed for the PCR amplification of mRNA for the full length GHRH-R and SVs were utilized. The PCR products were sequenced, and their specificity was confirmed. Nine ECs cancers (23%) expressed mRNA for SV1, three (7.7%) showed SV2 and eight (20.5%) revealed mRNA for SV4. The presence of SVs for GHRH-Rs could not be detected in any of the normal endometrial tissue specimens. The presence of specific, high affinity GHRH-Rs was also demonstrated in EC specimens using radioligand binding studies. Twenty-four of the investigated thirty-nine tumor samples (61.5%) and three of the seven corresponding normal endometrial tissues (42.9%) expressed mRNA for GHRH ligand. Our findings suggest the possible existence of an autocrine loop in EC based on GHRH and its tumoral SV receptors. The antiproliferative effects of GHRH antagonists on EC are likely to be exerted in part by the local SVs and GHRH system.

## 1. Introduction

The expression of splice variants (SVs) of the GHRH receptor (GHRH-R) has been found not only in the pituitary but in extrapituitary tissues, including human neoplasms [1,2,3,4]. cDNAs encoding for the four SVs of GHRH receptors were isolated and sequenced [5]. Based on these findings the cDNA sequence of SV1 was found to be similar to that of the full-length GHRH-R [5]. The first three exons were replaced in SV1 by a fragment of retained intron 3 possessing a new putative in-frame start codon; thus, the encoded N-terminal extracellular domain of SV1 is different from the pituitary-type GHRH-R protein [5]. SV1 appears to be the most functional isoform since SV2 encodes a GHRH isoform truncated after the second transmembrane domain, while SV3 and SV4 lack any transmembrane domains [5]. In support of this hypothesis, SV1 has been demonstrated to bind to GHRH and GHRH antagonists with high affinity and to mediate responses to GHRH in ligand-dependent and ligand-independent ways [6,7,8].

GHRH antagonists, in addition to their indirect antitumor effects through the GHRH- pituitary GH-hepatic insulin-like growth factor-I (IGF-I) axis, were shown to directly inhibit the proliferation of human cancer cell lines in vitro [9,10,11]. Moreover, these potent antineoplastic agents have been shown to suppress in vivo the growth of various human experimental cancers such as pancreatic [12], colorectal [13], prostate [14,15,16], breast [17,18,19] ovarian [20], renal [21] and lung [22,23,24] cancers; glioblastoma [25,26], osteosarcoma and Ewing sarcoma [27,28]; esophageal squamous cell carcinoma [29]; pleural mesothelioma [30]; as well as endometrial carcinoma [31]. In many of these tumors, the antiproliferative functions are mediated by SV-1. Although it has been known for more than 20 years that some cancers produce GHRH, it was only recently proposed that GHRH might function as an autocrine growth factor in neoplastic cells. mRNAs for GHRH or GHRH peptide were also found in surgical specimens of human endometrial, ovarian, breast and prostate cancers [1,2,32,33]. mRNAs encoding four SVs of GHRH-Rs, GHRH-R protein and specific high affinity binding sites for GHRH and its antagonistic analogs have been demonstrated in several experimental cancer models and specimens of human tumors [1,2,3,4,5,24,26,27,31,34,35,36,37]. Thus, the direct antiproliferative action of GHRH antagonists could be exerted by the disruption of an autocrine/paracrine loop of stimulation established by tumoral GHRH and its tumoral receptors.

Endometrial cancer (EC) is the sixth most common diagnosed malignancy in women [38]. Based on the estimates of the American Cancer Society, nearly 67,000 new cases of cancer of the uterus will be diagnosed, and approximately 13,000 women will die from cancers of the uterine body in the USA in 2021. Cancer of the uterine corpus is often referred to as endometrial cancer because more than 90% of cases occur in the endometrium (lining of the uterus) [39]. Based on Global Cancer Statistics 2020, about 417,000 new cases and 970,000 deaths of EC were confirmed worldwide [38].

In the last decade, a wide variety of treatment options was proposed as adjuvant therapies of EC. Chemotherapy, irradiation, use of immune checkpoint inhibitors and drugs aiming at molecular targets provide options for fighting EC. 

In earlier studies, the expression and role of the GHRH ligand was already investigated in some benign and malignant gynecologic conditions, including EC [32,33,40,41]. However, information on the splice variants of GHRH-Rs is rather limited. Fu et al. [42] demonstrated the expression of SV1 in endometriosis. The aim of the present study was to investigate the expression of GHRH and its tumoral receptors and the presence of GHRH-R SVs in primary human endometrial carcinoma samples and in corresponding benign endometrial tissues.

## 2. Results

### 2.1. Molecular Biology Analysis

New primers were designed for the PCR amplification of GHRH-R and SV1. PCR products were sequenced in both directions, and the specificity of the primers was confirmed. For GHRH-R, a 121 base-pair-long product was amplified from exon 1 to exon 2, which is present only in the full length receptor mRNA and absent in the splice variants. This product could be detected in none of the endometrial tumor specimens or normal endometrial tissues. However, as expected, the expression of mRNA for the full length GHRH-R was found in all five pituitary samples used as positive controls (data not shown). Accordingly, only the GHRH-R PCR product obtained from these samples was used for sequence analysis. In the case of the SV1 receptor variant, the 415-bp long PCR products (from intron 3, absent in the full length receptor; to exon 7, present only in SV1 and the full length receptor but not in the other variants) of the endometrial tumor samples were identical to that of the pituitaries.

**Figure 1:**
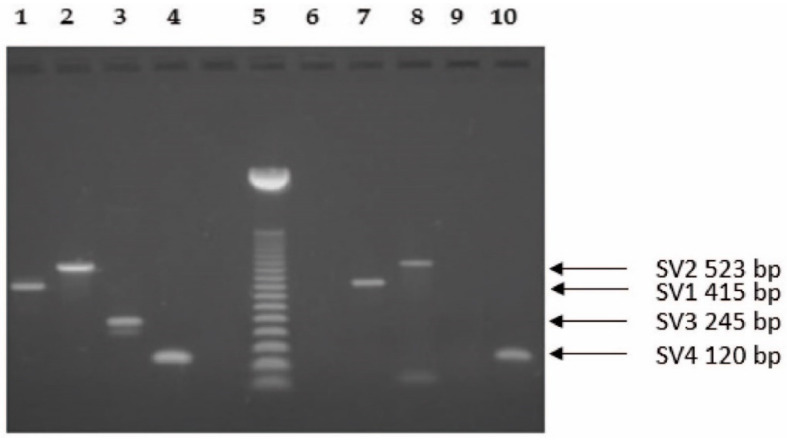
Representative RT-PCR analysis of the splice variants of GHRH receptor in a human pituitary sample used as a positive control for PCR (lanes 1–4) and in an endometrium carcinoma sample from patient 29 (lanes 7–10). Lane 5, 50-bp DNA Step Ladder. PCR products were of the expected sizes: SV1 415-bp, SV2 523-bp, SV3 245-bp (present only in the pituitary) and SV4 120-bp long. PCR products were separated by agarose gel electrophoresis and stained with ethidium bromide. Lane 6: negative template control.

The SV2, SV3 and SV4 splice variants were detected as 523-, 245- and 120-bp long PCR products, respectively [5]. Figure 1 shows the representative RT-PCR analysis of the splice variants. As a positive control, we have investigated five human pituitary tissues, all of which expressed the four splice variants and the full length GHRH-R.

**Figure 2:**
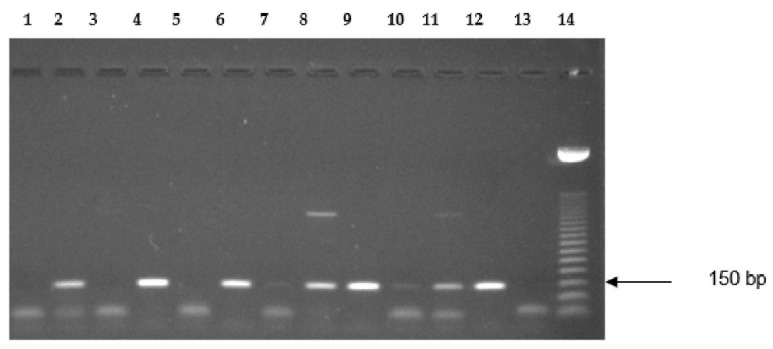
Representative RT-PCR analysis of the expression of mRNA for GHRH ligand. Lanes 1–11: representative endometrium tumor tissues; lane 12: positive control (human pituitary); lane 13: no template control; lane 14: 50-bp DNA ladder. PCR products were of the expected size of 150 base pairs. In samples with low or no expression, a primer dimer was also detected.

Twenty-four of the investigated thirty-nine tumor samples (61.5%) and three of the seven corresponding normal endometrial tissues (42.9%) expressed mRNA for GHRH ligand (Table 1, Figure 2). The expression of mRNA for GHRH was also detected in the five human pituitary tissues investigated (Figure 2).

In patients with endometrial carcinoma, SV1 is the most functional form in the view of a potential cancer therapy and could be shown in nine types of cancers (23%) (Figure 2, Table 1). The second most frequent variant was SV4, which was detected in 8 of 39 malignancies (20.5%). The incidence of SV2 could be observed only in three cancer specimen (7.7%), and the expression of SV3 variant was absent in the tumor samples (Figure 2, Table 1). The presence of the GHRH-R splice variants could not be revealed in any of the normal endometrial tissues investigated (Table 1).

Altogether, we were able to detect splice variants of GHRH-Rs in 14 of the 39 EC specimens (35.9%). The co-expression of mRNA for GHRH ligand and splice variants for GHRH-Rs was also found in 14 of 39 (35.9%) patients (Table 2). Our results show that all GHRH-R splice variant positive specimens expressed mRNA for the GHRH ligand. Ten of thirty-nine endometrial cancer specimens exhibited mRNA expression for GHRH but not for splice variants for GHRH-Rs. In five cases, only SV1or SV4 were expressed among the four splice variants of GHRH-Rs. In one case, SV1 and SV2 or SV1 and SV4 co-expression, and in other two cases, SV1, SV2 and SV4 co-expressions were observed. (Table 2).

### 2.2. Radioligand Binding Studies

The presence and binding characteristics of GHRH-Rs and specific binding of radioiodinated GHRH analog JV-1-42 to membrane homogenates of human EC samples were determined using radioreceptor assays. Of the eleven tumor specimens examined by ligand competition assays, nine samples (81.8%) showed GHRH binding (Table 3). The concentrations and binding affinities of GHRH-Rs in EC membranes were also investigated. The analyses of the displacement curves of [^125^I]JV-1-42 and the Scatchard plots of the specific binding data in the 9 receptor positive cancer specimens revealed that GHRH-Rs had a mean dissociation constant (K_d_) of 5.28 nM (range, 1.63 to 8.81 nM). The mean concentration of GHRH-Rs (maximal binding capacity, Bmax) was 385.0 fmol/mg membrane protein in crude membranes derived from human EC cells (range, 249.5 to 509.5 fmol/mg protein). Based on our receptor binding results, the one-site model could provide the best fit representing a single class of high affinity GHRH-Rs in human EC specimens. Biochemical specifications and parameters crucial to characterize specific binding sites were also defined. Thus, the in vitro receptor binding of [^125^I]JV-1-42 was detected to be specific, reversible, temperature dependent and time dependent, and linear with protein concentrations in the human endometrial tumor specimens examined (data not shown). The binding of radiolabeled JV-1-42 was displaced completely by increasing the concentrations (10^−12^–10^−6^ M) of hGHRH(1-44) or hGHRH(1-29)NH_2_, whereas none of the structurally and functionally different and unrelated peptides analyzed, such as somatostatin, luteinizing hormone-releasing hormone (LHRH), epidermal growth factor (EGF), [Tyr^4^]bombesin, and insulin-like growth factor I (IGF-I), inhibited the binding of radioiodinated JV-1-42 at concentrations as high as 1 µM (data not shown). Our results also showed that ligand binding was accompanied by the expression of mRNA for SV1 subtype of GHRH-Rs in all endometrial cancer specimens examined. A comparative analysis of the results of radioreceptor assays and SV1 subtype mRNA studies demonstrated that the expression of the SV1 subtype was 100% consistent with the presence of specific binding sites for GHRH antagonist [^125^I]JV-1-42 (Table 3).

In our study, no correlation was found among clinicopatholological features and receptor findings.

## 3. Discussion

Endometrial cancer is a major cause of morbidity and mortality for women worldwide, and it is the sixth most common malignancy among women [38,39]. Early stage EC has a favorable prognosis in general, but some women have aggressive malignancy because their tumors are high-grade, deeply invasive or consist of non-endometrioid cells (clear or papillary serous cells) and have a strong possibility for recurrence and death. Cases with EC are usually classified into two subtypes.

Based on Bokhman’s publication, we distinguish two main types of EC: Type I and Type II [43]. Type I endometrioid cancers are estrogen-dependent and arise from atypical endometrial hyperplasia. Thus, the excess of exogenous and endogenous estrogens has an important role in pathogenesis of Type I endometrial adenocarcinoma. Type II endometrioid cancers are less common, consist of more aggressive histological variants (i.e., clear-cell and serous carcinoma and uterine adenocarcinosarcoma), commonly occur in postmenopausal age and are associated with excessively high mortality [44]. Otherwise, Type II lesions are not related to long-lasting unopposed estrogen exposure. On the other hand, the molecular biology of EC became clearer in the past decade, leading to less morbid and minimally invasive surgical approaches and more routine utilizations of chemotherapy that have all made the outcomes of women with EC better. More efficient treatment modalities further improving survival and quality of life are strongly needed. 

Clinical trials of immune checkpoint inhibitors are in progress for advanced and recurrent endometrial cancer [45,46,47]. If a relationship with the genetic background of the administered population can be found and a good response rate is obtained, new treatments options can be introduced to replace standard treatment approaches [45,46,47].

Since 2018, the FDA has approved the use of immune checkpoint inhibitor, pembrolizumab (anti-PD-1, (programmed-cell death protein-Ligand 1)), for all solid tumors with defective DNA mismatched repairs. About 20–30% of patients with advanced EC can potentially benefit from its application [48,49]. Several studies suggested that chemotherapy not only may activate the immune system but also induce PD-L1 expression on cancer cells, which may result in more successful immunotherapy [45,49]. Ongoing observational studies try to improve the effect of immunotherapy (avelumab, atezolizumab and durvalumab) strategies with or without the combination of classic chemotherapy [49]. Other genomic changes and molecular markers in EC, such as hormone receptor status, could lead to more tailored therapy in the future. Preclinical and clinical investigations of targeted therapies suggest that some agents have efficacy for the treatment of EC [45].

It is widely accepted that GHRH acts as an autocrine/paracrine regulator for cancer cell proliferation [37,50]. Several splice variants (SVs) of the GHRH receptor have been isolated not only from pituitary but also from extrapituitary tissues, including human neoplasms [1,3,4,26,29,50]. Rekasi et al. found that the sequence of the main splice variant, SV1, is almost identical to that of the full-length (pituitary type) GHRH-R [5]. Opposed to pituitary type GHRH-R, the first three exons were replaced by a fragment of retained intron 3 possessing a new putative in-frame start codon in SV1, resulting only in a partial loss of the extracellular part of the pituitary type GHRH-R protein [5]. Based on the putative protein structure of SVs, SV1 appears to be the most probable functional receptor. Moreover, it has been demonstrated that SV1 binds GHRH and its antagonists with high affinity and mediates responses to GHRH [5]. In the present study, using RT-PCR, we demonstrated that mRNAs for GHRH and SVs, but not the pituitary type GHRH-R, are expressed in human EC tissues, suggesting the existence of an autocrine/paracrine GHRH loop.

In our work, we found that about one-third (35.9%) of EC specimens, but none of the normal endometrial tissues, were positive for one or more splice variants (SV1-4) of GHRH-R and 23% showed positivity for expression mRNA for SV-1. In an earlier study, 43% of endometrial cancer tissues were found to be positive for SV1 protein expression by immunohistochemistry [33]. This slight discrepancy could be explained by the fact that the antisera used for the detection of SV1 protein in this study was directed against the first 25 amino acids at the N terminus of the SV1 protein, which is also present in SV2 and SV4 subtypes. While SV1, SV2 and SV4 can be distinguished by size based on Western blotting, immunohistochemistry provides positive signals for all three GHRH-R isoforms. In addition, positive immunohistochemical signals were detected only in the cytoplasm of the epithelial cells of the glands of the endometrial adenocarcinomas but not on the cell’s surface. We found that the second most frequently expressed splice variant in our tissue series was SV4 (20.5%). The presence of the remaining two splice variants, SV2 and SV3, could be detected in only three or none of the samples, respectively. GHRH-R isoforms derived from SV3 and SV4 imply that they probably do not represent mature receptor proteins to be manifested on the cell’s surface. SV2, possessing the truncated N-terminal extracellular domain of SV1 but containing only two transmembrane domains, might be transported to the cell’s surface [5]. 

We could not detect mRNAs for pituitary type GHRH-R either in endometrium carcinoma or in normal endometrial tissues. In previous studies, the expression of pituitary GHRH-R was shown by real-time quantitative PCR in different cancer cell lines, including non-Hodgkin’s lymphoma, pancreatic cancer, glioblastoma and small-cell lung carcinoma, but the level of expression was low in extrapituitary normal tissues [3]. Our results are in agreement with previous findings, where the expression of classic pituitary type GHRH-R on different human tumor tissues could not be detected or was found to be less frequently present than SV1 [7,26,33,51].

In eleven cases, we were able to prepare crude membrane protein fractions for radioligand binding studies to demonstrate the presence of specific GHRH binding sites. Using ligand competition assays, we demonstrated the presence of specific, high affinity receptors for GHRH. Molecular biology analyses and radioligand binding studies clearly demonstrated that the expression of mRNA for SV1 subtype of GHRH-Rs was 100% consistent with the presence of specific receptors for radiolabeled GHRH analog JV-1-42. However, the expression of mRNA for the pituitary type of GHRH-Rs was not detected. It is also important to note that all receptor positive human EC specimens examined by ligand competition assay expressed a well-detectable amount of the SV1 GHRH-T gene. Furthermore, the PCR products for GHRH ligand were found in 24 of 39 (61.5%) human EC specimens. In 14 samples (35.9%), mRNA for both GHRH and GHRH-R splice variants was detected. While the most probable functional receptor splice variant SV1 was present in only 23% of the EC specimens investigated, the GHRH ligand could be detected in more than 60% of tumoral and 40% of normal endometrial tissues. In an earlier study, GHRH mRNA was detectable in normal endometrium and EC; however, no changes in endometrial GHRH mRNA were shown between normal and neoplastic tissues obtained from the same patient. However, the levels were higher than those found in myometrial tissues obtained from other patients from benign gynecologic diseases [40]. Thus, it was suggested that GHRH may promote endometrial proliferation and be involved in the pathogenesis of EC and endometriosis [40]. 

In another study investigating the presence of GHRH and SV1 in normal mouse tissues, a group of tissues was examined, including endometrium, and expressed GHRH but not its receptor SV1 [52]. The authors assumed that the presence of GHRH in these tissues is not coincidental but is physiologically important and may be consistent with the paracrine/endocrine action of neurohormons and extrapituitary actions of GHRH being mediated not only by SV1, but by other receptor(s) as well [52]. 

Previous studies have shown that GHRH antagonists, such as MZ-J-7-118, MZ-5-156 and JMR-132, inhibited the growth of human experimental ECs both in vitro and in vivo [31,53,54]. The beneficial oncological effects of these antagonists in experimental cancer treatment can be attributed to the suppression of pituitary-hepatic IGF-I axis and the direct inhibition through the binding of GHRH antagonists to pituitary GHRH-R and/or their splice variants present on tumors [36,50,55]. A recent study also demonstrated a mechanism by which GHRH-R antagonists such as MIA-602 target SV1 and inhibit the tumor growth of esophageal aquamous cell carcinoma mediated by SV1 [29]. Their findings suggest that SV1 is a hypoxia-induced oncogenic promoter that can be a potential target of GHRH-R antagonists [29].

Based on the evidence that GHRH antagonists were able to suppress experimental tumor growth and that a subset of EC expressed receptors for GHRH, the application of powerful new GHRH antagonists could be useful for the treatment of this type of malignancy. However, further studies were needed to validate this assumption.

In the future, we would like to expand our investigation and try to collect a reasonable number of human EC specimens to further study and analyze the expression of GHRH-Rs in such human tissues. These studies may provide novel quantitative data on the mRNA and protein levels of GHRH-Rs and their splice variants. From these results, we would be able to predict the potential response of the patients to GHRH-R-based therapy.

## 4. Materials and Methods

### 4.1. Tissue Samples

Human endometrial carcinoma specimens from 39 patients (mean age 62 years; range 28–82 years) who underwent surgical removal of their uterus at the Department of Obstetrics and Gynecology, Faculty of Medicine, University of Debrecen, were investigated. Approximately 5–20 mm^3^ of tissue samples of the uterus removed during staging surgery were used. Histopathological examinations of each specimen were undertaken to confirm the presence of endometrial carcinoma before molecular biology studies. There were 28 endometrioid (71.8%) and 11 papillary serous (28.2%) adenocarcinomas. Among patients with endometrioid adenocarcinoma, five had grade 1, twenty had grade 2 and three had grade 3 diseases. Among patients with the papillary serous subtype, three had grade 1, five had grade 2 and three had grade 3 cancers (Table 4). Normal endometrial tissues were available in seven cases. Tissue samples were frozen and stored at −80 °C until total RNA isolation and membrane preparations were performed. The collection and the use of these specimens and normal human pituitary samples in our studies was conducted in accordance with the Declaration of Helsinki and approved by the local institutional ethics committee named Regional Institutional Ethics Committee, Clinical Center, University of Debrecen (DERKEB/IKEB 2284-004). Informed consent was obtained from all patients. Five normal human pituitary reference samples used as positive controls were collected in an anonymous fashion from the paraffin tissue-archives of autopsy cases at the Department of Pathology, Faculty of Medicine, University of Debrecen.

### 4.2. RNA Isolation

Tissue samples were homogenized with a Mikro-Dismembrator-U (SartoriusB. Braun Biotech, Melsungen, Germany) and were used for RNA extraction with a Nucleospin Total RNA Isolation Kit (Macherey-Nagel, Düren, Germany). RNA concentration and purity were determined using the Nanodrop ND-1000 UV Spectrophotometer (Nanodrop Technologies, Wilmington, DE, USA). 

### 4.3. RT-PCR

One µg of total RNA was reverse transcribed to cDNA with MMLV Reverse Transcriptase and oligo(dT)15 (Promega Co, Madison, WI, USA) according to the manufacturer’s instructions. Primers for GHRH-R, sense 5′-CACGTCTTCTGCGTGTTGAG-3′ (exon 1) and antisense 5′-GCATCTCCTCTGCTGCTTGT-3′ (exon 2), for SV1, sense 5′-GGAAGGAGTTGTGGCTAGAGAG-3′ (intron 3) and antisense 5′-GTCATGGTGGCGAAATGG-3′ (exon 7) were designed using primer3_www.cgi v 0.2 program [56]. PCR products were sequenced on an ABI-PRISM 3130 Genetic Analyzer (Applied Biosystems, Foster City, CA, USA) in both directions to confirm the specificity of the primers. Gene-specific primers for β-actin housekeeping gene, GHRH ligand, SV2, SV3 and SV4 splice variants were used as described previously [56,57].

For β-actin, GHRH-R, GHRH and SV1 genes the PCR reaction mix contained 1 × PCR Buffer, 1U Taq Polymerase (Invitrogen, NY, USA), 1.5 mM MgCl_2_, 0.3 μM of each primer (Invitrogen), 200 μM of each dNTP (Fermentas, Germany) and 1.0 μL cDNA template in a final volume of 25 μL.

After denaturation (3 min at 94 °C), cDNA was amplified for 45 cycles (45 s at 94 °C; 30 s at 62 °C and 90 s at 72 °C). β-actin was amplified with 30 cycles. Then, a final elongation step of 72 °C 10 min was applied, and finally, the samples were cooled down to 4 °C.

For SV2, SV3 and SV4 splice variants, the PCR reaction mix contained 1 × PCR Buffer, 1.25 U TruStart Taq Polymerase (Fermentas), 3 mM (SV2, SV4) or 4 mM (SV3) MgCl_2_, 0.4 μM (SV2, SV4) or 0.5 μM (SV3) of each primer; 300 μM (SV2) or 200 μM (SV3, SV4) of each dNTP; and 1.5 μL cDNA in a final volume of 25 μL.

After denaturation and enzyme activation (3 min at 95 °C), cDNA was amplified for 45 cycles (30 s at 95 °C; 30 s at 63 °C and 60 s at 72 °C). Then, a final elongation step of 72 °C 5 min was applied, and finally, the samples were cooled down to 4 °C. The PCR products were separated by electrophoresis on 2% agarose gel stained with ethidium bromide.

### 4.4. Preparation of Membranes and Radioligand Binding Studies

Radioiodinated derivatives of GHRH antagonist JV-1-42 were prepared by the chloramine-T method, as previously described [1] with some minor modifications. The preparation of tumor cell membranes from human EC samples for the receptor binding studies was performed as reported previously [1]. Briefly, the human cancer specimens were homogenized in 50 mmol/L Tris-HCl buffer (pH 7.4) and supplemented with protease inhibitors (0.25 mmol/L phenylmethylsulfonylfluoride, 2 μg/mL pepstatin A, and 0.4% aprotinin) using an Ultra-Turrax tissue homogenizer (IKA Works, Wilmington, NC, USA); then, the crude membrane fraction was prepared as described [1] and stored at −70 °C until investigated in vitro. Protein concentrations were determined by the method of Bradford. GHRH-R binding assays were carried out, as reported in detail, using in vitro ligand competition assays based on the binding of [^125^I]JV-1-42 as radioligands to membrane fractions of human EC specimens [1]. GHRH antagonist JV-1-42 and [^125^I]JV-1-42 as radioligand were well-characterized previously and showed high-affinity binding to rat and human pituitaries and human renal, prostate, breast and other cancers [1,10,17,50]. The high affinity binding of radioiodinated JV-1-42 to SV1 was also demonstrated and reported previously [1]. In brief, membrane homogenates containing 50–160 μg protein were incubated in duplicate or triplicate with 60,000–80,000 cpm [^125^I]JV-1-42 and increasing concentrations (10^−12^–10^−6^ mol/L) of nonradioactive peptides as competitors in a total volume of 300 μL binding buffer (50 mmol/L Tris-HCl, 5 mmol/L EDTA, 5 mmol/L MgCl_2_, 1% BSA and 30 μg/mL bacitracin, pH 7.4) supplemented with protease inhibitors, as mentioned above. After 1 h of incubation and the separation, the final pellet containing the receptor bound fraction was counted in a γ-counter [1]. The LIGAND-PC computerized curve-fitting software of Munson and Rodbard was used to determine the type of receptor binding, dissociation constant (Kd) and maximal binding capacity of the receptors (Bmax). Due to the limited amounts of membrane protein fractions, the receptor binding of GHRH was examined in only 11 specimens.

## Figures and Tables

**Table 1:** Expression of mRNA for the full length GHRH receptor, its splice variants and the GHRH ligand in 39 human endometrial carcinoma samples and 7 normal human endometrial tissue samples.

Gene	Positive/Total Sample Size (Tumor)	%
GHRH-R	0/39	0
GHRH	24/39	61.5
SV1	9/39	23.0
SV2	3/39	7.7
SV3	0/39	0
SV4	8/39	20.5
Gene	Positive/Total Sample Size (Normal)	%
GHRH-R	0/7	0
GHRH	3/7	42.9
SV1	0/7	0
SV2	0/7	0
SV3	0/7	0
SV4	0/7	0

**Table 2:** Clinicopathological features and mRNA expression pattern of receptors for GHRH, and GHRH ligand in endometrial cancer specimens positive for any of the GHRH receptor splice variants.

Patient No	Age at Diagnosis	Histology *	Grade	Stage	GHRH-R	GHRH	SV1	SV2	SV3	SV4
6	52	E	2	I/b	−	+	+	+	−	−
8	49	P-S	1	I/b	−	+	+	−	−	−
9	65	E	2	I/a	−	+	−	−	−	+
12	67	E	1	I/a	−	+	+	+	−	+
13	76	E	1	I/b	−	+	−	−	−	+
14	66	E	2	I/b	−	+	−	−	−	+
16	67	P-S	1	I/b	−	+	+	−	−	+
20	72	E	2	I/b	−	+	−	−	−	+
24	53	P-S	1	II/b	−	+	+	−	−	−
29	48	E	2	III/c	−	+	+	+	−	+
30	43	P-S	3	III/c	−	+	−	−	−	+
32	70	E	2	II/a	−	+	+	−	−	−
36	63	E	2	III/c	−	+	+	−	−	−
37	48	P-S	2	II/a	−	+	+	−	−	−

* P-S: papillary serous adenocarcinoma; E: endometrioid endometrial carcinoma.

**Table 3:** Expression of mRNA for SV1 and binding characteristics of GHRH receptors in 11 human endometrial cancer specimens.

Patient Number	mRNA for SV1	Kd (nM)	Bmax (fmol/mg Protein)
6	+	8.81	509.5
7	−	−	−
8	+	4.02	297.9
12	+	4.78	276.0
16	+	2.17	474.7
24	+	8.74	486.9
29	+	4.77	415.3
32	+	1.63	482.2
34	−	−	−
36	+	5.77	249.5
37	+	6.86	273.0

**Table 4:** Clinicopathological features (FIGO stage, grade and histologic subtype) of 39 patients with endometrial cancer (endometrioid and papillary-serous adenocarcinoma).

FIGO Stage	Grade 1	Grade 2	Grade 3	Total
IA	0	4	1	5
IB	6	9	2	17
IC	0	3	1	4
Total stage I	6	16	4	26
IIA	0	3	0	3
IIB	2	2	1	5
Total stage II	2	5	1	8
IIIA	0	2	0	2
IIIC	0	2	1	3
Total stage III	0	4	1	5
Total	8	25	6	39
Endometrioid subtype	5	20	3	28
papillary-serous subtype	3	5	3	11

Summary of the clinical data for the 39 patients with endometrial cancer (endometrioid, *n* = 28; papillary-serous, *n* = 11). Specimens from patients with a diagnosis of endometrioid adenocarcinoma were graded: well differentiated (grade 1), moderately differentiated (grade 2) or poorly differentiated (grade 3).

## Data Availability

The data presented in this study are available on request from the corresponding author.

## Sample Availability

Samples of the compounds are available from the authors.

## References

[1] Halmos G., Schally A.V., Czompoly T., Krupa M., Varga J.L., Rekasi Z. (2002). Expression of growth hormone-releasing hormone and its receptor splice variants in human prostate cancer. J. Clin. Endocrinol. Metab..

[2] Chatzistamou I., Schally A.V., Kiaris H., Politi E., Varga J., Kanellis G., Kalofoutis A., Pafiti A., Koutselini H. (2004). Immunohistochemical detection of GHRH and its receptor splice variant 1 in primary human breast cancers. Eur. J. Endocrinol..

[3] Havt A., Schally A.V., Halmos G., Varga J.L., Toller G.L., Horvath J.E., Szepeshazi K., Köster F., Kovitz K., Groot K. (2005). The expression of the pituitary growth hormone-releasing hormone receptor and its splice variants in normal and neoplastic human tissues. Proc. Natl. Acad. Sci. USA.

[4] Freddi S., Arnaldi G., Fazioli F., Scarpelli M., Appolloni G., Mancini T., Kola B., Bertagna X., Mantero F., Collu R. (2005). Expression of growth hormone-releasing hormone receptor splicing variants in human primary adrenocortical tumours. Clin. Endocrinol..

[5] Rekasi Z., Czompoly T., Schally A.V., Halmos G. (2000). Isolation and sequencing of cDNAs for splice variants of growth hormone-releasing hormone receptors from human cancers. Proc. Natl. Acad. Sci. USA.

[6] Barabutis N., Tsellou E., Schally A.V., Kouloheri S., Kalofoutis A., Kiaris H. (2007). Stimulation of proliferation of MCF-7 breast cancer cells by a transfected splice variant of growth hormone-releasing hormone receptor. Proc. Natl. Acad. Sci. USA.

[7] Kiaris H., Chatzistamou I., Schally A.V., Halmos G., Varga J.L., Koutselini H., Kalofoutis A. (2003). Ligand-dependent and -independent effects of splice variant 1 of growth hormone-releasing hormone receptor. Proc. Natl. Acad. Sci. USA.

[8] Kiaris H., Schally A.V., Busto R., Halmos G., Artavanis-Tsakonas S., Varga J.L. (2002). Expression of a splice variant of the receptor for GHRH in 3T3 fibroblasts activates cell proliferation responses to GHRH analogs. Proc. Natl. Acad. Sci. USA.

[9] Csernus V.J., Schally A.V., Kiaris H., Armatis P. (1999). Inhibition of growth, production of insulin-like growth factor-II (IGF-II), and expression of IGF-II mRNA of human cancer cell lines by antagonistic analogs of growth hormone-releasing hormone in vitro. Proc. Natl. Acad. Sci. USA.

[10] Rekasi Z., Varga J.L., Schally A.V., Halmos G., Armatis P., Groot K., Czompoly T. (2000). Antagonists of growth hormone-releasing hormone and vasoactive intestinal peptide inhibit tumor proliferation by different mechanisms: Evidence from in vitro studies on human prostatic and pancreatic cancers. Endocrinology.

[11] Rekasi Z., Varga J.L., Schally A.V., Plonowski A., Halmos G., Csernus B., Armatis P., Groot K. (2001). Antiproliferative actions of growth hormone-releasing hormone antagonists on MiaPaCa-2 human pancreatic cancer cells involve cAMP independent pathways. Peptides.

[12] Szepeshazi K., Schally A.V., Groot K., Armatis P., Hebert F., Halmos G. (2000). Antagonists of growth hormone-releasing hormone (GH-RH) inhibit in vivo proliferation of experimental pancreatic cancers and decrease IGF-II levels in tumours. Eur. J. Cancer.

[13] Szepeshazi K., Schally A.V., Groot K., Armatis P., Halmos G., Herbert F., Szende B., Varga J.L., Zarandi M. (2000). Antagonists of growth hormone-releasing hormone (GH-RH) inhibit IGF-II production and growth of HT-29 human colon cancers. Br. J. Cancer.

[14] Jungwirth A., Schally A.V., Pinski J., Halmos G., Groot K., Armatis P., Vadillo-Buenfil M. (1997). Inhibition of in vivo proliferation of androgen-independent prostate cancers by an antagonist of growth hormone-releasing hormone. Br. J. Cancer.

[15] Lamharzi N., Schally A.V., Koppán M., Groot K. (1998). Growth hormone-releasing hormone antagonist MZ-5-156 inhibits growth of DU-145 human androgen-independent prostate carcinoma in nude mice and suppresses the levels and mRNA expression of insulin-like growth factor II in tumors. Proc. Natl. Acad. Sci. USA.

[16] Letsch M., Schally A.V., Busto R., Bajo A.M., Varga J.L. (2003). Growth hormone-releasing hormone (GHRH) antagonists inhibit the proliferation of androgen-dependent and -independent prostate cancers. Proc. Natl. Acad. Sci. USA.

[17] Kahán Z., Varga J.L., Schally A.V., Rékási Z., Armatis P., Chatzistamou L., Czömpöly T., Halmos G. (2000). Antagonists of growth hormone-releasing hormone arrest the growth of MDA-MB-468 estrogen-independent human breast cancers in nude mice. Breast Cancer Res. Treat..

[18] Chatzistamou I., Schally A.V., Varga J.L., Groot K., Busto R., Armatis P., Halmos G. (2001). Inhibition of growth and metastases of MDA-MB-435 human estrogen-independent breast cancers by an antagonist of growth hormone-releasing hormone. Anti-Cancer Drugs.

[19] Khanlari M., Schally A.V., Block N.L., Nadji M. (2018). Expression of GHRH-R, a Potentially Targetable Biomarker, in Triple-negative Breast Cancer. Appl. Immunohistochem. Mol. Morphol..

[20] Chatzistamou I., Schally A.V., Varga J.L., Groot K., Armatis P., Bajo A.M. (2001). Inhibition of growth and reduction in tumorigenicity of UCI-107 ovarian cancer by antagonists of growth hormone-releasing hormone and vasoactive intestinal peptide. J. Cancer Res. Clin. Oncol..

[21] Jungwirth A., Schally A.V., Pinski J., Groot K., Armatis P., Halmos G. (1997). Growth hormone-releasing hormone antagonist MZ-4-71 inhibits in vivo proliferation of Caki-I renal adenocarcinoma. Proc. Natl. Acad. Sci. USA.

[22] Pinski J., Schally A., Jungwirth A., Groot K., Halmos G., Armatis P., Zarandi M., Vadillobuenfil M. (1996). Inhibition of growth of human small cell and non-small cell lung carcinomas by antagonists of growth hormone-releasing hormone (GH-RH). Int. J. Oncol..

[23] Kiaris H., Schally A.V., Varga J.L., Groot K., Armatis P. (1999). Growth hormone-releasing hormone: An autocrine growth factor for small cell lung carcinoma. Proc. Natl. Acad. Sci. USA.

[24] Szereday Z., Schally A.V., Varga J.L., Kanashiro C.A., Hebert F., Armatis P., Groot K., Szepeshazi K., Halmos G., Busto R. (2003). Antagonists of growth hormone-releasing hormone inhibit the proliferation of experimental non-small cell lung carcinoma. Cancer Res..

[25] Kiaris H., Schally A.V., Varga J.L. (2000). Antagonists of growth hormone-releasing hormone inhibit the growth of U-87MG human glioblastoma in nude mice. Neoplasia.

[26] Mezey G., Treszl A., Schally A.V., Block N.L., Vízkeleti L., Juhász A., Klekner A., Nagy J., Balázs M., Halmos G. (2014). Prognosis in human glioblastoma based on expression of ligand growth hormone-releasing hormone, pituitary-type growth hormone-releasing hormone receptor, its splicing variant receptors, EGF receptor and PTEN genes. J. Cancer Res. Clin. Oncol..

[27] Braczkowski R., Schally A.V., Plonowski A., Varga J.L., Groot K., Krupa M., Armatis P. (2002). Inhibition of proliferation in human MNNG/HOS osteosarcoma and SK-ES-1 Ewing sarcoma cell lines in vitro and in vivo by antagonists of growth hormone-releasing hormone: Effects on insulin-like growth factor II. Cancer.

[28] Pinski J., Schally A.V., Groot K., Halmos G., Szepeshazi K., Zarandi M., Armatis P. (1995). Inhibition of growth of human osteosarcomas by antagonists of growth hormone-releasing hormone. J. Natl. Cancer Inst..

[29] Xiong X., Ke X., Wang L., Yao Z., Guo Y., Zhang X., Chen Y., Pang C.P., Schally A.V., Zhang H. (2020). Splice variant of growth hormone-releasing hormone receptor drives esophageal squamous cell carcinoma conferring a therapeutic target. Proc. Natl. Acad. Sci. USA.

[30] Villanova T., Gesmundo I., Audrito V., Vitale N., Silvagno F., Musuraca C., Righi L., Libener R., Riganti C., Bironzo P. (2019). Antagonists of growth hormone-releasing hormone (GHRH) inhibit the growth of human malignant pleural mesothelioma. Proc. Natl. Acad. Sci. USA.

[31] Engel J.B., Keller G., Schally A.V., Toller G.L., Groot K., Havt A., Armatis P., Zarandi M., Varga J.L., Halmos G. (2005). Inhibition of growth of experimental human endometrial cancer by an antagonist of growth hormone-releasing hormone. J. Clin. Endocrinol. Metab..

[32] Kahán Z., Arencibia J.M., Csernus V.J., Groot K., Kineman R.D., Robinson W.R., Schally A.V. (1999). Expression of growth hormone-releasing hormone (GHRH) messenger ribonucleic acid and the presence of biologically active GHRH in human breast, endometrial, and ovarian cancers. J. Clin. Endocrinol. Metab..

[33] Chatzistamou I., Schally A.V., Pafiti A., Kiaris H., Koutselini H. (2002). Expression of growth hormone-releasing hormone in human primary endometrial carcinomas. Eur. J. Endocrinol..

[34] Busto R., Schally A.V., Varga J.L., Garcia-Fernandez M.O., Groot K., Armatis P., Szepeshazi K. (2002). The expression of growth hormone-releasing hormone (GHRH) and splice variants of its receptor in human gastroenteropancreatic carcinomas. Proc. Natl. Acad. Sci. USA.

[35] Plonowski A., Schally A.V., Busto R., Krupa M., Varga J.L., Halmos G. (2002). Expression of growth hormone-releasing hormone (GHRH) and splice variants of GHRH receptors in human experimental prostate cancers. Peptides.

[36] Schally A.V., Wang H., He J., Cai R., Sha W., Popovics P., Perez R., Vidaurre I., Zhang X. (2018). Agonists of growth hormone-releasing hormone (GHRH) inhibit human experimental cancers in vivo by down-regulating receptors for GHRH. Proc. Natl. Acad. Sci. USA.

[37] Schally A.V., Zhang X., Cai R., Hare J.M., Granata R., Bartoli M. (2019). Actions and Potential Therapeutic Applications of Growth Hormone-Releasing Hormone Agonists. Endocrinology.

[38] Sung H., Ferlay J., Siegel R.L., Laversanne M., Soerjomataram I., Jemal A., Bray F. (2021). Global Cancer Statistics 2020: GLOBOCAN Estimates of Incidence and Mortality Worldwide for 36 Cancers in 185 Countries. CA Cancer J. Clin..

[39] Siegel R.L., Miller K.D., Fuchs H.E., Jemal A. (2021). Cancer Statistics, 2021. CA Cancer J. Clin..

[40] Khorram O., Garthwaite M., Grosen E., Golos T. (2001). Human uterine and ovarian expression of growth hormone-releasing hormone messenger RNA in benign and malignant gynecologic conditions. Fertil. Steril..

[41] Kővári B., Vranic S., Marchio C., Sapino A., Cserni G. (2017). The expression of GHRH and its receptors in breast carcinomas with apocrine differentiation-further evidence of the presence of a GHRH pathway in these tumors. Hum. Pathol..

[42] Fu L., Osuga Y., Yano T., Takemura Y., Morimoto C., Hirota Y., Schally A.V., Taketani Y. (2009). Expression and possible implication of growth hormone-releasing hormone receptor splice variant 1 in endometriosis. Fertil. Steril..

[43] Bokhman J.V. (1983). Two pathogenetic types of endometrial carcinoma. Gynecol. Oncol..

[44] Wright J.D., Barrena Medel N.I., Sehouli J., Fujiwara K., Herzog T.J. (2012). Contemporary management of endometrial cancer. Lancet.

[45] Arend R.C., Jones B.A., Martinez A., Goodfellow P. (2018). Endometrial cancer: Molecular markers and management of advanced stage disease. Gynecol. Oncol..

[46] Mitamura T., Dong P., Ihira K., Kudo M., Watari H. (2019). Molecular-targeted therapies and precision medicine for endometrial cancer. Jpn. J. Clin. Oncol..

[47] Aoki Y., Kanao H., Wang X., Yunokawa M., Omatsu K., Fusegi A., Takeshima N. (2020). Adjuvant treatment of endometrial cancer today. Jpn. J. Clin. Oncol..

[48] Lu K.H., Broaddus R.R. (2020). Endometrial Cancer. N. Engl. J. Med..

[49] Paleari L., Pesce S., Rutigliani M., Greppi M., Obino V., Gorlero F., Vellone V.G., Marcenaro E. (2021). New Insights into Endometrial Cancer. Cancers.

[50] Schally A.V., Varga J.L., Engel J.B. (2008). Antagonists of growth-hormone-releasing hormone: An emerging new therapy for cancer. Nat. Clin. Pract. Endocrinol. Metab..

[51] Köster F., Engel J.B., Schally A.V., Hönig A., Schröer A., Seitz S., Hohla F., Ortmann O., Diedrich K., Buchholz S. (2009). Triple-negative breast cancers express receptors for growth hormone-releasing hormone (GHRH) and respond to GHRH antagonists with growth inhibition. Breast Cancer Res. Treat..

[52] Christodoulou C., Schally A.V., Chatzistamou I., Kondi-Pafiti A., Lamnissou K., Kouloheri S., Kalofoutis A., Kiaris H. (2006). Expression of growth hormone-releasing hormone (GHRH) and splice variant of GHRH receptors in normal mouse tissues. Regul. Pept..

[53] Zhao L., Yano T., Osuga Y., Nakagawa S., Oishi H., Wada-Hiraike O., Tang X., Yano N., Kugu K., Schally A.V. (2008). Cellular mechanisms of growth inhibition of human endometrial cancer cell line by an antagonist of growth hormone-releasing hormone. Int. J. Oncol..

[54] Wu H.M., Schally A.V., Cheng J.C., Zarandi M., Varga J., Leung P.C. (2010). Growth hormone-releasing hormone antagonist induces apoptosis of human endometrial cancer cells through PKCδ-mediated activation of p53/p21. Cancer Lett..

[55] Kovács M., Schally A.V., Varga J.L., Zarándi M. (2008). Endocrine and antineoplastic actions of growth hormone-releasing hormone antagonists. Curr. Med. Chem..

[56] Rozen S., Skaletsky H. (2000). Primer3 on the WWW for general users and for biologist programmers. Methods Mol. Biol..

[57] Øvstebø R., Haug K.B., Lande K., Kierulf P. (2003). PCR-based calibration curves for studies of quantitative gene expression in human monocytes: Development and evaluation. Clin. Chem..

